# Association of Optic Neuritis with *CYP4F2* Gene Single Nucleotide Polymorphism and IL-17A Concentration

**DOI:** 10.1155/2018/1686297

**Published:** 2018-03-15

**Authors:** Mantas Banevicius, Alvita Vilkeviciute, Brigita Glebauskiene, Loresa Kriauciuniene, Rasa Liutkeviciene

**Affiliations:** ^1^Department of Ophthalmology, Medical Academy, Lithuanian University of Health Sciences, Eiveniu 2, LT-50161 Kaunas, Lithuania; ^2^Neuroscience Institute, Medical Academy, Lithuanian University of Health Sciences, Eiveniu 2, LT-50161 Kaunas, Lithuania

## Abstract

**Background:**

The aetiology and pathophysiology of optic neuritis (ON) is not absolutely clear but genetic and inflammatory factors may be also involved in its development. The aim of the present study was to determine the influence of single nucleotide polymorphism (SNP) of *CYP4F2* (rs1558139) and serum levels of IL-17A on ON development.

**Materials and Methods:**

Forty patients with ON and 164 control subjects were evaluated. Patients were divided by gender, also ON patients were divided into two subgroups: ON with and without multiple sclerosis (MS). *CYP4F2* rs1558139 was genotyped using real-time PCR. Serum IL-17A levels were measured using ELISA IL-17A kits.

**Results:**

We found that A/A genotype of *CYP4F2* rs1558139 was statistically significantly more frequent in men with ON and MS than in women: 57.1% versus 0%, *p* = 0.009. Also, allele A was statistically significantly more frequent in men with ON and MS than in women: 71.4% versus 37.5%, *p* = 0.044. Serum levels of IL-17A were higher in ON group than in control group: (median, IQR): 20.55 pg/ml, 30.66 pg/ml versus 8.97 pg/ml, 6.24 pg/ml, *p* < 0.001.

**Conclusion:**

The higher IL-17A levels were found to be associated with ON, while allele A at rs1558139 was associated only with ON with MS in male patients.

## 1. Introduction

Optic neuritis (ON) represents demyelinating inflammation of the optic nerve, which slows or blocks the transmission of signals to and from the brain. Many genetic heterogeneous conditions may cause ON. A gradual recovery of visual acuity over time is a characteristic of typical ON, which is characterized by painful, usually monocular visual loss with decreased visual acuity, defects of the visual field and colour vision [1]. The most common cause is acute demyelinating ON, which is associated with multiple sclerosis (MS) as its first symptom [2]. MS is most often diagnosed in young adults and women are affected more than men by a ratio of 2 : 1 [2, 3]. ON is also more frequent in young adults aged 18–45 [1] and the ratio of female preponderance is approximately 3 : 1 [3]. ON is associated with the loss of axons, nerve impulse blockade, or ganglion cell death [4]. Disruption of nerve impulses by the inflammatory process leads to type IV delayed hypersensitivity reaction. When activated peripheral T lymphocytes release cytokines and other inflammatory mediators that can move through the blood-brain barrier and cause decomposition of myelin, nerve cell death, and axonal degeneration [5]. Disruption of the blood-brain barrier leads to T lymphocytes and inflammatory mediators accessing the central nervous system and indicates beginning of optic neuritis [6–8]. The causes of ON are not known. It is believed that the ON is caused by genetic and environmental factors [9]. Typical ON is often associated with multiple sclerosis (MS), causing axon loss [2]. Atypical ON maybe caused by bacterial, fungal, or viral infections and other inflammatory and autoimmune diseases [10]. Genetic and inflammatory factors may also take part in ON development. It is known that *CYP4F2* gene is found in neurons and glial cells, where the enzyme encoded by this gene metabolises arachidonic acid, helping to maintain the structure of cell membranes in the hippocampus [11] and protecting the brain against oxidative stress [12]. This gene also activates enzymes involved in the growth and regeneration of neurons [13]. Impaired arachidonic acid metabolism is associated with ON, MS, Alzheimer's disease, and bipolar disorder [14]. *CYP4F2*-encoded enzyme reduces arachidonic acid metabolites as well as a number of prostaglandins, which are involved in various inflammatory processes. Also this enzyme is involved in drug metabolism and the synthesis of cholesterol, steroids, fatty acids, and other lipids [15]. It has been shown that this gene single nucleotide polymorphism (SNP) is associated with the development of hypertension [16], cerebral infarction [17], myocardial infarction [18–21], and metabolic syndrome [22]. Also, it has been found that SNP plays a role in the processes of anticoagulant drug metabolism [23, 24].

Another inflammatory factor, associated with neuroinflammatory diseases, is interleukin - 17A (IL-17A). IL - 17A directly affects neurons, but it can also have an effect on the nerves through the signals to the satellite and immune cells. In the central nervous system, IL-17A is associated with a wide range of neuropathological disorders (MS, epilepsy episodes of ischemic brain disorders). IL-17A, acting on spinal nerve roots and the spinal cord, contributes to neuropathic and inflammatory pain promotion through pain receptors. Also, IL-17A is very important to damaged sympathetic axons which innervate the cornea regeneration and growth. In a number of chronic noninfectious diseases involving inflammatory response, the elevated IL-17A concentrations are found [25]. The aim of our research was to find the association of IL-17A and *CYP4F2* rs1558139 gene polymorphism with optic neuritis.

## 2. Materials and Methods

The permission (number BEC-LSMU (ER-19)) to undertake the study was obtained from Kaunas Regional Biomedical Research Ethics Committee. The study was conducted in the Department of Ophthalmology of the Hospital of Lithuanian University of Health Sciences and Neuroscience Institute, Lithuanian University of Health Sciences. Study participants comprised of 40 subjects with a diagnosis of optic neuritis and 164 persons from the control group. All patients with an attack of ON who were admitted to the Hospital of Lithuanian University of Health Sciences Ophthalmology department between the 1st of January 2012 and the 1st of February 2015 were included in our study. The inclusion criteria for subjects with optic neuritis were as follows: (1) patients with the first attack of an acute ON episode; (2) participation consent; (3) neurologist consultation; and (4) conducted neurological examination for MS (MS diagnosis was established according to the widely accepted and revised McDonald criteria (2005)). The exclusion criteria for subjects were as follows: (1) other diseases of the optic nerve and (2) systemic illnesses (diabetes mellitus, oncological diseases, systemic tissue disorders, chronic infectious diseases, and conditions after organ or tissue transplantation). The inclusion criteria for healthy patients were (1) no ophthalmological eye disorders found on detailed ophthalmological evaluation; (2) detailed general clinical examination of the patients; and (3) participation consent. The exclusion criteria for healthy patients were (1) Any eye disorders and (2) Any general therapeutic disorders. The reference group was made of healthy subjects, who were admitted to the Hospital of Lithuanian University of Health Sciences Ophthalmology Department for preventive ophthalmological evaluation, considering the patient's age and gender in the optic neuritis group. Therefore, the medians of the patient age of the control group and the optic neuritis group did not differ statistically significant (*p* < 0.05). Demographic data of the study subjects are presented in Table 1.

### 2.1. SNP Selection

According to literature data, rs2108622 is a mostly often studied CYP variant. CYP rs1558139 variant (NM_001082.4:c.919-446C>T) was already evaluated in patients with ischemic stroke. In our study, this SNP was selected according to a Chinese scientist group study [19]. There are 225 SNPs for the human *CYP4F2* gene listed in the National Center for Biotechnology Information SNP database Build 126 (http://www.ncbi.nlm.nih.gov/SNP). Authors screened the data for the Tag SNPs on the International HapMap Project website (http://www.hapmap.org/index.html.ja), using a cutoff level of *r*^2^ ≥ 0.5, and for the minor allele frequency, we used a cutoff level of ≥0.1. According to the above criterion, we selected rs1558139 for this gene as well, and found out that

1. our genotyped SNP rs1558139 was in very low linkage disequilibrium with rs2108622 which was reported to be linked to ischemic stroke (*r*^2^ = 0.009);
2. additionally, the SNP Function Prediction tool developed by Xu and Taylor [26] and available online at the SNPinfo Web Server (https://snpinfo.niehs.nih.gov/) reported that the intron variant rs1558139 have regulatory potential function;
3. on the other hand, there still exist a limitation of papers with functional annotation of associations of SNPs and optic neuritis.

### 2.2. DNA Extraction and Genotyping

The DNA extraction and analysis of the gene polymorphism of *CYP4F2* (G1347A; rs1558139) was carried out in the Laboratory of Ophthalmology, Neuroscience Institute of LUHS. DNA was extracted from 200 *μ*L venous blood (white blood cells) using a DNA purification kit based on the magnetic beads method (MagJET Genomic DNA Kit, Thermo Scientific) according to the manufacturer's recommendations. The genotyping of *CYP4F2* (G1347A; rs1558139 was carried out using the real-time polymerase chain reaction (PCR) method. Single nucleotide polymorphisms were determined using TaqMan® Drug Metabolism assays (Thermo Scientific). The genotyping was performed using a Rotor-Gene Q real-time PCR quantification system (Qiagen, USA). The real-time PCR reagents (2X TaqMan Universal Master Mix, TaqMan Drug Metabolism assay, and nuclease-free water) were stored at −20°C and thawed at room temperature. Then reagents were centrifuged (10,000 rpm) and stored in an ice tub. The appropriate real-time PCR mixture of *CYP4F2* (G1347A) was prepared for determining single nucleotide polymorphism. The PCR reaction mixture (9 *μ*L) was poured into each of the 72 wells of the Rotor-Disc, and then 1 *μ*L of matrix DNA of the samples (~10 ng) and 1 *μ*L of negative control (−K) were added. The Allelic Discrimination program was used during the real-time PCR. The assay was continued following the manual provided by the manufacturer (http://www.qiagen.com, Allelic Discrimination). After the Allelic Discrimination program was completed, the genotyping results were obtained. The program determined the individual genotypes according to the fluorescence intensity rate of the different detectors (VIC and FAM).

### 2.3. Assessment of Serum Interleukin-17A Levels

Serum levels of IL-17A were evaluated using an ELISA kit (“Thermo Fisher Scientific Human IL17A ELISA Kit”) designed to measure human IL-17A in serum.

### 2.4. Statistical Analysis

Statistical analysis was performed using the SPSS/W 20.0 software (Statistical Package for the Social Sciences for Windows Inc., Chicago, Illinois, USA). The data are presented as absolute numbers with percentages in brackets, average values, and standard deviations (SD). Hardy–Weinberg analysis was performed to compare the observed and expected frequencies of rs1558139 genotypes using the *χ*^2^ test in all groups. The distribution of the rs1558139 SNP genotypes in the ON and control groups was compared using the *χ*^2^ test or the Fisher's exact test. Binomial logistic regression analysis was performed to estimate the impact of genotypes on AMD development. Odds ratios and 95% confidence intervals are presented. The selection of the best genetic model was based on the Akaike information criterion (AIC); therefore, the best genetic models were those with the lowest AIC values. R studio version 3.4.1 (RStudio Team (2017). RStudio: Integrated Development for R. RStudio Inc., Boston, MA, URL http://www.rstudio.com/) was used for IL-17A concentration analysis. The D'Agostino-Pearson test was used to check the data for normal distribution. The nonparametric data were presented as median and interquartile range (IQR; 25th to 75th percentile).The Mann–Whitney test was used for comparison between these data. When the distribution of the data was parametric, the unpaired Student's *t*-test was used and the results were presented as mean and standard deviation (SD). Differences were considered statistically significant for all tests when *p* < 0.05.

## 3. Results

There were two groups in our study. The first included 40 ON patients: 13 men (32.5%) and 27 women (67.5%) and the median age was 35. The control group consisted of 164 healthy persons: 32 men (19.5%) and 132 (80.5%) women with a median age of 44. Demographic characteristics are shown in Table 1.

### 3.1. Analysis of Association of *CYP4F2* rs1558139 with Optic Neuritis

Rs1558139 genotype distribution for both studied groups was at Hardy–Weinberg equilibrium (*p* > 0.05). Analysis of *CYP4F2* rs1558139 genotype and allele distribution between ON patients and healthy controls did not reveal any statistically significant differences (Table 2).

Binomial logistic regression analysis was performed. However, it did not show any statistically significant differences (Table 3).

The detailed analysis was performed to analyze genotype distribution by gender, but it did not show any differences either. For the further analysis, there were two new groups of ON patients formed: the ON patients without MS and the ON patients with MS. *CYP4F2* rs1558139 genotype distributions only showed differences when the analysis was performed by gender. Rs1558139 A/A genotype was statistically significantly more frequent in men than in women: 57.1% versus 0%, respectively, *p* = 0.009 (Table 4). Also, allele A was statistically significantly more frequent in men than in women: 71.4% versus 37.5%, respectively, *p* = 0.044 (Table 4).

Binomial logistic regression was performed, but it did not reveal any statistically significant differences.

### 3.2. IL-17A Concentration Measurements

**Figure 1:**
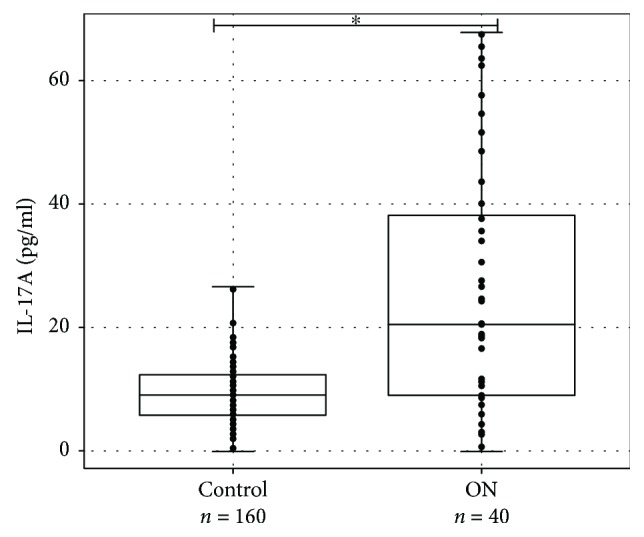
IL-17A concentration (pg/ml) in serum of ON patients and healthy controls. IL-17A levels (pg/ml) in serum of ON patient versus healthy controls are presented as *box-*and-*whisker plots* with the median and IQR and individual dots. Mann–Whitney *U* test was used to assess the differences of IL-17A concentration between ON patients and control groups. ^∗^*p* < 0.001.

IL-17A was detected in serum samples from all patients and healthy controls. According to the D'Agostino-Pearson test, IL-17A values in serum did not follow the normal distribution and the result was expressed by median and interquartile range (IQR). IL-17A serum concentration was significantly higher in ON patients than in healthy controls (median, IQR: 20.55 pg/ml, 30.66 pg/ml versus 8.97 pg/ml, 6.24 pg/ml, respectively, *p* < 0.001) (Figure 1).

### 3.3. IL-17A Concentration in ON Patients with and without Multiple Sclerosis

**Figure 2:**
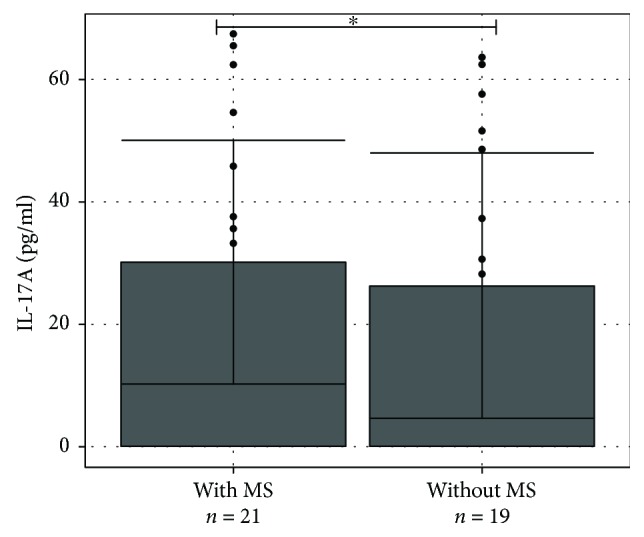
IL-17A concentration (pg/ml) in serum of ON patients with and without MS. IL-17A levels (pg/ml) in serum of ON patients with and without MS are presented as *bar plots* with the mean and SD and individual dots. Student's *t-*test was used to assess the differences of IL-17A concentration among ON without MS and ON with MS groups. ^∗^*p* = 0.544.

Further analysis was performed to evaluate differences of IL-17A level between ON patients with and without MS. According to the D'Agostino-Pearson test, IL-17A values in serum followed the normal distribution and the result was expressed by mean standard deviation (SD). Student's *t*-test showed that IL-17A concentration in studied groups was normally spread (*p* < 0.05), therefore comparing the concentration of IL-17A between these two groups, the unpaired Student's *t*-test was used. Comparing concentration of cytokine IL-17A in blood serum of ON patients with or without MS, statistically significant differences were not found (average (SD): 26.19 (21.55) pg/ml versus 30.21 (19.96) pg/ml, respectively, *p* = 0.544) (Figure 2).

### 3.4. Analysis of Association between Serum IL-17A Levels and Genotypes of *CYP4F2* rs1558139

**Figure 3:**
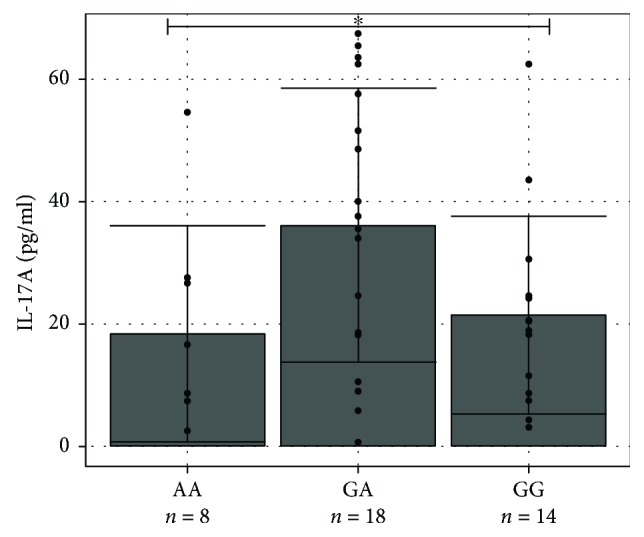
IL-17A concentration (pg/ml) in serum of different genotype groups. IL-17A levels (pg/ml) in serum of ON patients by genotype are presented as *bar plots* with the mean and SD and individual dots. *One-way ANOVA test* was used to assess the differences of IL-17A concentration between groups of ON patients with and without MS. ^∗^*p* = 0.603.

We compared IL-17A levels in serum between all genotype groups of ON patients as well. IL-17A concentration in the three genotype groups (GG, GA, and AA) was normally distributed (*p* > 0.05). The one-way ANOVA analysis did not reveal any differences between IL-17A concentration by genotypes (average (SD): 21.35 (16.13) pg/ml versus 36.20 (22.35) pg/ml versus 18.33 (17.65) pg/ml, respectively, *p* = 0.603) (Figure 3).

## 4. Discussion

Our study showed that *CYP4F2* gene rs1558139 polymorphism did not reveal any differences in the distribution of G/G, G/A, and A/A genotypes between groups (35.0% versus 45.0% versus 20.0% in patients with optic neuritis, 30.5% versus 47.0% versus 22.5% in control group, resp.). However, the group of patients with optic neuritis without MS had statistically significant differences between genders (*p* < 0.05). Rs1558139 A/A genotype was statistically significantly more frequent in males than females: 57.1% versus 0%, *p* = 0.009, but it must be repeated with a larger sample of the study for the reliability of the results. Patients with acute demyelinating ON are young adults, usually without any other health problems. Female preponderance in observed, with a ratio of approximately 3 : 1. ON is seen more commonly in Caucasians and quite rarely in black populations. Incidence of ON is eight times higher in white northern Europeans than blacks and Asians [27]. Animal experiments have revealed that the expression of a number of CYP (cytochrome P450 enzymes) is sex dependent [28, 29]. Cytochrome P450 (CYP) enzymes are also involved in estrogen metabolism and many are regulated by estrogens. These genes may thus be of relevance to gender-specific differences in lung cancer risk, particularly in early-onset lung cancer, where a high proportion of women is observed [30].We also hypothesised that one of the SNPs in *CYP4F2* (rs1558139) is important in pathogenetic mechanisms of optic neuritis in females. On the other hand, sex dependence in human was found in few other studies: Fava et al. [20] found that V433M mutation in CYP4F2 gene was associated with cerebral infarction in male patients and the study [31] based on Japanese population found that G allele at rs2108622 was more frequent in male patients than in the controls. Haplotype analysis revealed that TCG haplotypes composed by rs3093135-rs1558139-rs2108622 is a risk factor for cerebral infarction in males [18].

An inflammation is one of the key players in pathogenesis of optic neuritis [10]. The data on CYP activity during inflammation process is scare and inconsistent. Haberfeld reported that inflammation suppresses CYP activity [32], but Gilroy et al. stated that levels of CYP 450 enzyme-derived oxylipins are elevated as the expression of *CYP4F2*, *CYP4F3*, and *CYP4A* family enzymes is increased during inflammation [33]. We have not found similar studies investigating *CYP4F2* gene polymorphisms in patients with optic neuritis. Fu and coauthors found that *CYP4F2* rs1558139 polymorphism in combination with other polymorphisms of this gene, which form haplotypes, are related to cardiac and cerebral vascular diseases [18]. In 2015, Lan et al. showed that four polymorphisms (rs7528684, rs945635, rs3761959, and rs2282284) in FCRL3 gene can increase the risk for neuromyelitis optica (NMO) [34]. FCRL3_3 (rs7528684) polymorphism, widely studied in earlier studies, showed that this polymorphism is a characteristic of many autoimmune diseases [35–37]. Recently it was found that rs703842 and rs10876994 in *CYP27B1* gene that encodes a vitamin D metabolising protein are related to neurological demyelinating diseases in a Chinese population. Statistically significant differences were found between genotype distributions of rs703842 (*p* = 0.013) and rs10876994 (*p* = 0.001) polymorphisms comparing patients with their control group [38]. Several studies determined that rs1016140 in *CD58* gene was associated with reduced T cell activity [39, 40]. It was found that the G allele at rs1016140 was associated with an increased risk of CNS diseases. It is believed that an increase in T cell activity depends on the G allele and may result in higher CNS inflammation frequency, which in turn facilitates access of aquaporin 4 (AQP4), a water carrier membrane antibody, into the CNS and ultimately leads to the spread of optic neuritis. Further studies are needed to confirm the role of rs1016140 [41].

Interleukin-17A (IL-17A) participates in the pathogenesis of several immune and inflammatory diseases and upregulates expression of numerous inflammation-related genes [42]. Our research revealed that IL-17A concentration in blood serum was statistically significantly higher for patients with ON in comparison with control group (median, IQR): 18.33 (7.41–37.6) pg/ml versus 8.19 (5.85–11.31) pg/ml, *p* < 0.001), but IL-17A concentration in blood serum of patients with ON with or without MS was not statistically significantly different (average (SD): 27.76 (24.13) pg/ml versus 31.57 (21.89) pg/ml, respectively; *p* = 0.641). There are no studies analysing association of IL-17A concentration with ON, but we think that cytokines are very important for the development of optic neuritis.

Pathogenic mechanisms of inflammatory Th17 cells, releasing a large amount of interleukin-17 (IL-17) and IL-23 and other cytokines are already widely studied [43]. TGF-*β* cytokine is also adopted in the framework of the differentiation of T cells associated with MS and NMO [44, 45]. Another study with mice autoimmune encephalomyelitis models showed that Th17, IL-6, and IL-17 concentration increases may be associated with autoimmune disorders of central nervous system (CNS). In the experimental study of mice models, a strong relationship was found between IL-17 and Th17 cells [46, 47]. In particular, it showed that sharp differences in IL-17 concentration increase in a mouse model of autoimmune encephalomyelitis [48]. IL-17A association with optic neuritis was also proved in experimental model by Knier et al. [49].

NMO is associated with astrocytic water channel aquaporin-4 (AQP4) antibodies, which is believed to contribute to the pathogenesis of optic nerve inflammation and causes toxic reactions and immune cell infiltration. It is believed that AQP4 antibodies may be considered a reliable marker in distinguishing NMO from MS. AQP4 antibody concentrations appear to be strongly correlated with IL-6 and IL-8 concentrations [48]. In addition, IL-17 and IL-23 levels are elevated in patients with NMO, compared with controls [50].

### 4.1. Strengths and Limitations

This is the first study to examine the relationship of *CYP4F2* rs1558139 and IL-17A levels with ON and MS. For the first time, *CYP4F2* rs1558139 AA genotype was associated with ON in males with MS, and for the first time, the higher IL-17A levels were determined to be associated with ON. On the other hand, because of a large number of SNPs, it is required to make replication studies in the future, particularly with bigger sample size, to confirm the association between SNPs and ON development.

## 5. Conclusions

We revealed *CYP4F2* rs1558139 AA genotype association with ON and MS in male. According to statistical analysis, the higher IL-17A levels were determined to be associated with inflammation processes while ON. These findings suggest new biological markers for the diagnostics of ON, but further studies with a larger number of patients, however, are necessary in order to better understand of ON development.

## Figures and Tables

**Table 1:** Demographic characteristics of patients with optic neuritis (ON) and control group subjects.

Characteristics	ON group (*n* = 40)	Control group (*n* = 164)	*p* value
Gender			
Females, *n* (%)	27 (67.5)	132 (80.5)	0.618
Males, *n* (%)	13 (32.5)	32 (19.5)	
Age, year (min/max/median)	18/57/35	19/65/44	0.735
ON patients without MS^∗^	19	—	—
Females	13	—	—
Males	6	—	—
ON patients with MS	21	—	—
Females	14	—	—
Males	7	—	—

^∗^MS: multiple sclerosis.

**Table 2:** Frequency of *CYP4F2* rs1558139 genotypes and alleles in patients with optic neuritis (ON) and in control group.

Gene	Genotype/allele	Frequency (%)				
		Control group *n* (%) *n* = 164	*p* HWE	ON group *n* (%) *n* = 40	*p* HWE	*p* value
*CYP4F2* rs1558139	Genotype					
	G/G	50 (30.5)	0.481	14 (35)	0.616	*χ* ^2^ = 0.331
	G/A	77 (47)		18 (45)		*p* = 0.848
	A/A	37 (22.5)		8 (20)		
	Total	164 (100)		40 (100)		
	Allele					
	G	177 (54.0)		46 (57.5)		*p* = 0.569
	A	151 (46.0)		34 (42.5)		

ON: optic neuritis; *p* value: significance level (alpha = 0.05); *p* value HWE: significance level (alpha = 0.05) by Hardy–Weinberg equilibrium.

**Table 3:** *CYP4F2* rs1558139 binomial logistic regression analysis in patients with optic neuritis (ON) and in control group.

Model	Genotype	OR (CI 95%)	*p* value	AIC
Codominant	G/A	0.835 (0.381–1.828)	0.652	207.599
	A/A	0.722 (0.294–2.031)	0.600	
Dominant	G/A + A/A	0.815 (0.393–1.690)	0.582	205.963
Recessive	A/A	0.858 (0.364–2.022)	0.726	205.802
Overdominant	G/A	0.924 (0.462–1.851)	0.824	205.877
Additive	A allele	0.873 (0.540–1.411)	0.580	205.619

ON: optic neuritis; OR: odd ratio; CI: confidence interval; *p* value: significance level (alpha = 0.05); AIC: Akaike information criterion.

**Table 4:** Frequency of *CYP4F2* rs1558139 genotypes and alleles in the groups of optic neuritis (ON) patients with and without MS by gender.

Gene	Genotype/allele	Frequency (%)					
		ON without MS group *n* (%)		*P* value	ON with MS group *n* (%)		*P* value
		Women *n* = 12	Men *n* = 7		Women *n* = 15	Men *n* = 6	
*CYP4F2* rs1558139	Genotype						
	G/G	3 (27)	1 (14.3)	1.000	6 (32.1)	4 (66.7)	0.362
	G/A	9 (75)	2 (28.6)	0.074	5 (53.6)	2 (33.3)	1.000
	A/A	0 (0)	4 (57.1)	0.009	4 (14.3)	0 (0)	0.281
	Total	12 (100)	7 (100)		15 (100)	6 (100)	
	Allele						
	G	15 (62.5)	4 (28.6)	0.044	17 (56.7)	10 (83.33)	0.158
	A	9 (37.5)	10 (71.4)		13 (43.3)	2 (16.67)	

ON: optic neuritis; MS: multiple sclerosis: *p* value: significance level (alpha = 0.05).

## Abbreviations

ON: Optic neuritis
CYP: Cytochrome P450 enzyme
IL: Interleukin
MS: Multiple sclerosis
SNP: Single nucleotide polymorphism
IQR: Interquartile range
SD: Standard deviation
HWE: Hardy–Weinberg equilibrium
NMO: Neuromyelitis optica
FCRL3: Fc receptor-like 3 gene
CNS: Central nerve system
AQP4: Aquaporin 4 protein
TGF-*β*: Transforming growth factor beta
Th: T helper.
